# The Need for Sensory-Friendly “Zones”: Learning From Youth on the Autism Spectrum, Their Families, and Autistic Mentors Using a Participatory Approach

**DOI:** 10.3389/fpsyg.2022.883331

**Published:** 2022-06-15

**Authors:** Marc-André Clément, Keven Lee, Melissa Park, Anabel Sinn, Natalie Miyake

**Affiliations:** ^1^School of Physical and Occupational Therapy, Faculty of Medicine and Health Sciences, McGill University, Montreal, QC, Canada; ^2^Centre de Recherche Interdisciplinaire en Réadaptation, Montreal, QC, Canada; ^3^Lady Davis Institute, Montreal, QC, Canada; ^4^addtothenoise, Los Angeles, CA, United States; ^5^WIAIH, Pointe-Claire, QC, Canada

**Keywords:** autism, occupational participation, photovoice, experience, universal design (UD), built environment, bodily-sensing, sociality

## Abstract

**Introduction:**

Differences in sensory processing were linked to a diagnosis of autism spectrum disorder (ASD) before its inclusion as a core characteristic in the revised DSM-V. Yet, research focused on sensory processing and meaningful participation of children and youth with ASD remains relatively scarce. Although refinement of the International Classification of Functioning and Disability (ICF) relies on first-person accounts, longitudinal studies that foreground sensory experiences and its impact on involvement in a life situation from first-person perspectives are largely missing from this body of research.

**Objectives:**

In this sub-study, we drew from a longitudinal participatory research project consisting of two separately funded studies with children and youth with ASD and their families between 2014 and 2021. The participatory project used photovoice (PV) methods to identify the primary concerns related to socio-spatial exclusion (PV-1) and the action steps needed to redress them (PV-2). The objective of this sub-study was to understand what really mattered to children with autism, their parents, autistic youth and an adult mentor to consider how their experiential knowledge could deepen understanding of meaningful participation.

**Materials and Methods:**

We used an overarching narrative phenomenological and aesthetic theoretical framework to focus data analysis on the bodily sensing experiences related to significant moments or events, followed by an inductive thematic analysis of what mattered about those moments.

**Results:**

The topical areas of concern that emerged from analyses were: (1) the relationship between sensory experiences and mental health (*motion madness*); (2) the indivisibility or layering of sensory and social experiences (*squishing and squeezing*); (3) the impact when “tricks” to stay involved are categorically misunderstood (*When you don’t respond in the correct way*), and (4) how care and consideration of others can lead to innovative solutions for inclusion (*I can’t be the only one*). Listening to the bodily-sensing experiences of children with ASD, autistic youth and adults, and their families in their own terms has implications for remapping the ICF and envisioning sensory curb-cuts to access, initiate and sustain occupational participation for all.

## Introduction

Sensory processing, such as hyper- and hypo-reactivity to sensory stimuli in the environment, is included as a core characteristic of autism spectrum disorder (ASD) in the revised Diagnostic and Statistical Manual of Mental Disorders (DSM-V) under the category of restricted, repetitive patterns of behaviors, interests, or activities (APA, 2013). Early on, research with specific attention to hyper-responsiveness to sensory input demonstrated that infants, toddlers, and children diagnosed with ASD—aged between 5 and 83 months—have different sensory processing patterns (Baranek et al., 2007). More recently, a meta-analysis of over five decades of research demonstrated that persons with ASD have significant differences in patterns of sensory responsivity than other comparison groups, such as typically developing or other neurological diagnoses (Ben-Sasson et al., 2019). Since the publication of the revised DSM-V and the focus on the sensory processing of persons with ASD, research demonstrates a steady increase of 1.2% of the diagnosis for children and youth between 1 and 17 years of age (Diallo et al., 2018). Yet, surprisingly, there is relatively little research focused on the sensory processing of children and youth with ASD and participation.

The research on children and youth with ASD and participation is also relatively scarce. For example, Askari et al. (2015) found only 16 articles in their scoping review of ASD and participation in leisure activities outside of school. Although the scoping review used the domains in the International Classification of Functioning, Disability, and Health (ICF) to categorize results, only three quantitative cross-sectional studies mentioned sensory processing (Tomchek and Dunn, 2007; Hochhauser and Engel-Yeger, 2010; Reynolds et al., 2011). In contrast, a systematic review (Ismael et al., 2018) that explicitly used Dunn’s sensory processing framework (Dunn, 2001; Dunn, 2007) to focus on the participation of children with ASD yielded seven studies that found that sensory processing had a significant impact on participation in everyday life.

Participation, defined as “involvement in life situations” (World Health Organization, 2002), is conceptualized in the ICF as being restricted by body functions and structures (impairment), activities (limitation), and environmental and personal factors. Although the ICF was ground-breaking in bringing together the terms of biomedicine and the social model of disability, it has long been critiqued for its absence of ways to conceptualize agency, intentionality, subjective meaning, and the dynamic interaction between persons and environments, as well as how the ICF can support actual interventions (Jahiel, 2015). Both Jahiel’s (2015) structural reformulation of the ICF and Askari et al.’s (2015) scoping review of participation intersect in a mutual critique of the static nature of the ICF from two different perspectives. From a conceptual perspective, the ICF is only a “snapshot” of what its codes/qualifiers can capture at a given moment of time (Jahiel, 2015, p. 19), while methodologically, the quantitative, cross-sectional studies of participation underscore the need for more longitudinal studies (Askari et al., 2015). Further, as Jahiel (2015) astutely pointed out, “Very few [research discussions] have dealt with the “how” question” (p. 19); that is, how can research on participation support its actualization?

Jahiel (2015) also marked how interviews and focus groups contribute to the development of instruments to measure the subjective meaning of participation, while also noting that first-person perspectives on how the environment significantly impacts on participation has not yielded the same results. For example, autists’ autobiographies describe how their “extraordinarily heightened senses” are intimately related to experiences of what the geographer and critical autism studies scholar Davidson (2010) called barriers to “socio-spatial inclusion” that are further exacerbated by lack of understanding by non-autistic others (p. 309). Yet, research on sensory processing and participation has rarely included the experiences of children and youth with ASD from their first-person perspectives (e.g., see Kirby et al., 2015 as a rare exception). Instead, qualitative studies on the participation of children and youth with ASD have primarily relied on parental perspectives (Rios and Scharoun Benson, 2020, see also Howell and Pierson, 2010; Lam et al., 2010; and Thompson and Emira, 2011 in Askari et al., 2015), and only one study focused on the impact of the sensory environment on the participation of children with ASD (Pfeiffer et al., 2017).

Thus, in this article, we draw from a longitudinal, participatory research project that consisted of two separately funded studies with children and youth with ASD and their families that took place between 2014 and 2021. We used photovoice methods, with the overarching aim to identify the primary concerns (barriers and facilitators) to socio-spatial inclusion (Photovoice-1) and action steps to redress them (Photovoice-2). In the sub-study that we report on here, our objective was to understand what really mattered to autistic youth about their bodily-sensing experiences to consider how their experiential knowledge could deepen understanding of participation from first-person perspectives.

## Materials and Methods

This qualitative sub-study draws key exemplars from a longitudinal participatory project, consisting of two studies funded through separate mechanisms across a 7-year period. Participatory research is an approach in which key stakeholders who become co-researchers identify key concerns and develop actions to address those concerns (Jagosh et al., 2015). In our project, we used photovoice, an approach to participatory research in which persons use and-or make images (typically photographs, mini-videos) to share experiences in response to a question of direct concern to them (Wang and Burris, 1997). Although participatory approaches originate in social critical approaches, researchers’ underlying epistemologies and conceptual or theoretical frameworks often shape the actual process (e.g., see Asaba et al., 2014). Arguably, as Asaba and Suarez-Balcazar (2018) point out, participatory approaches “gained momentum in times when scholars were examining methodological approaches to address participation and health disparities,” although there remain differing levels of community or participant engagement (p. 309). We describe the conceptual frameworks and differing levels of engagement of autistic persons and their parents in the two studies below.

### Recruitment and Participants

For recruitment of the first photovoice study (PV-1), a parent who was a key stakeholder (NM) informed families in her network about the project. If they were interested in participating, researchers met them, and completed a formal consent process. Six children between the age of 5–12 years old (1 female:5 males) and their parents (5 females:1 male) were recruited. Recruitment for the second photovoice study (PV-2) consisted of re-engaging families from PV-1 who were still interested and through personal networks for older autistic youth. In total, 10 autistic persons (3 females:7 males) along with four parents were recruited. In PV-2, the youth, older youth and adult identified their own gender. Three of the youth with autism and two parents were in both studies (see Table 1).

**TABLE 1:** Recruitment results for PV1 and PV2.

Participant	Gender	Role	Participation in PV1	Age at time of PV1	Participation in PV2	Age at time of PV2
Leo	M	Participant	✓	6		
Phillipe	M	Participant	✓	6		
Marco	M	Participant	✓	7		
Karl	M	Participant	✓	11		
Breanna	F	Participant/Co-researcher	✓	10	✓	15
Paul	M	Participant/Co-researcher	✓	11	✓	17
Victor	M	Participant/Co-researcher	✓	12	✓	17
Keith	M	Co-researcher			✓	17
David	M	Co-researcher			✓	17
Cassandra	F	Older youth consultant			✓	22
Sophie	F	Older youth consultant			✓	26
Joshua	M	Older youth consultant			✓	24
Samuel	M	Older youth consultant			✓	27
Casey	M	Adult mentor			✓	42

Sensory processing questionnaires were used in both studies to foster discussions around sensory experiences. We report on them here to situate their experiences. In PV-1, we used the Short Sensory Profile (SSP) (McIntosh et al., 1999), a 38-item caregiver questionnaire that describes children’s sensory processing patterns. The scoring from the SSP is expressed as a range from “typical performance” to “definite difference” across seven sensory subscales (see Table 2). In PV-2, we used the Adolescent and Adult Sensory Profile (AASP) (Brown et al., 2001). Based on Dunn’s Model of Sensory Processing, the AASP is a self-reported sensory processing pattern questionnaire in which the scores describe the individual’s neurological threshold and behavioral response continuum across four quadrants (Brown et al., 2001). The scoring within each quadrant ranges from “much less than most” to “much more than most people” (see Table 3).

**TABLE 2:** Scores from the Short Sensory Profile (SSP) of the autistic individuals implicated in the participatory photovoice project through 2014–2016 (PV1).

	Scores from the short sensory profile							
	Tactile sensitivity	Taste/smell sensitivity	Movement sensitivity	Underresponsive/Seeks sensation	Auditory filtering	Low energy/weak	Visual/auditory sensitivity	Total score
Leo	PD	TP	TP	DD	DD	TP	DD	DD
Philippe	TP	DD	TP	DD	DD	TP	PD	DD
Marco	DD	TP	DD	DD	DD	DD	DD	DD
Breanna	DD	DD	DD	DD	DD	DD	DD	DD
Paul	DD	DD	TP	DD	DD	DD	PD	DD
Victor	PD	PD	TP	DD	PD	DD	PD	DD

*TP, Typical Performance; PD, Probable Difference; DD, Definite Difference.*

**TABLE 3:** Scores from the Adolescent and Adult Sensory Profile (AASP) of the autistic individuals implicated in the participatory photovoice project 2018–2021 (PV2).

	Low registration	Sensation seeking	Sensory sensitivities	Sensory avoiding
Breanna	++	=	+	+
Paul	=	–	=	++
Victor	+	=	++	++
Keith	+	– –	++	++
David	–	–	=	+
Cassandra	+	+	++	++
Sophie	U/A	U/A	U/A	U/A
Joshua	U/A	U/A	U/A	U/A
Samuel	=	=	=	+
Casey	++	–	++	++

*=, similar to most people; +, more than most people; ++, much more than most people; –, less than most people; – –, much less than most people; U/A, data unavailable.*

The research settings were based in community centers for youth with disabilities in the greater Montreal area in Québec, Canada. Both studies were approved by a university ethics review board.

### Conceptual Framework

The longitudinal participatory photovoice project used narrative phenomenological and aesthetic conceptual frameworks. The narrative phenomenological framework focused data collection and analysis on significant experiences or events from first-person (i.e., I, we) and multiple perspectives (Mattingly, 2010). Events are the memorable moments that stand out in experience (Dewey, 1934; Jackson, 2005), which often emerge during intersubjective moments in which there is something at stake (Jackson, 1998). The aesthetic conceptual framework heightened attention to the tight entanglement of bodily-sensing experiences, the narrative forms used to represent them (e.g., metaphor), and considerations of the good (Park, 2010). Further we integrated Mattingly’s (2019) critical phenomenology 2.0 during data analysis of this sub-study on sensory processing and participation to mark “perplexing particulars”. A perplexing particular is “an encounter that not only surprises in the sense of striking unexpectedly, but also eludes explanation” (p. 429). As a form of experience, perplexing particulars provide a critical edge by asking researchers to reconsider established categories or assumptions from the first-person perspective of those with whom they conduct research. Finally, during the representation of data, we used philosophical-literary terms, such as bodily-sensing, to foreground the *experiences* related to sensory processing from a first-person perspective rather than positivist-biomedical terms that focus attention on sensory processing from a neurological perspective (modulation, regulation) and-or categories (auditory, olfactory, vestibular-proprioceptive, visual, and etc.) (i.e., see Park, 2008).

### Data Collection/Analysis

The primary method for data collection across the longitudinal participatory project was the group meeting to discuss images taken. These meetings were structured as “collective narratives,” a method in narrative phenomenology in which each person has a chance to speak without interruption (Mattingly, 2010). We also conducted individual narrative interviews (Mattingly and Lawlor, 2000) when persons were unable to attend the groups. All collective narratives were held in the meeting rooms of community-based organizations familiar to the families, and recorded, de-identified, and transcribed verbatim.

#### Photovoice 1

The first study emerged from a small research project initiated by researchers at a local children’s hospital to understand the resources needed by parents of children with ASD. However, the parents’ primary concerns were more related to their exclusion from—and lack of awareness about the sensory challenges in—their local communities. Subsequently, the first photovoice study was a pilot project using ethnographic methods and participatory approaches to understand what really mattered to families with children with ASD, sensory experiences and social-spatial exclusion from their first-person perspectives using photovoice (Park, 2014–2016). All families were compensated for their time.

We conducted two collective narratives to understand what really mattered to the parents and children, using the following questions: “What are your favorite activities, including any that take place in public spaces?” and “What would you like to do with your children in public spaces that you cannot do at this time?” (120 min/group). We then organized a collective narrative in which photographs and videos were shared in response to the question, “What are barriers to doing what they would like to do in public spaces?” (180 min/group). The children (including the youngest at age 6) and their parents contributed photographs. Most of the stories were told by the parents, with two of the older children sharing their experiences at the third group. We conducted one interview (90 min/interview) (Total: 480 min).

#### Photovoice 2

The second study explicitly used a participatory approach in which the initial grant application included a parent of a youth with autism from PV-1 who was the project leader, a sustainable designer who guided the public facing initiatives, and a researcher who guided pragmatics related to academia, such as the grant writing/submission, ethics, and management of data collection/analysis (Park et al., 2018–2021). All autistic youth and parents who engaged in the study were considered co-researchers and compensated for their time. The aim was to collaboratively develop mechanisms to create more inclusive social-spatial communities by starting with the everyday places frequented by the youth—either those in which they felt excluded or those they identified as being “ideal” from a sensory perspective.

We organized four collective narratives (120 min/group). The participatory process consisted of the following steps. First, the youth contributed photographs and stories about their experiences in the specific places they envisioned change, with parents adding occasional anecdotes. Second, action steps emerged during and from these shared experiences. For example, at the first collective narrative, shared stories about sensory experiences in spaces they had recently frequented led to discussions about what objective measures could be used for others to understand their sensory challenges in those spaces. Third, individuals in the group found tools which they vetted and modified to map and measure the actual sound levels and sensory experiences in those spaces. Thus, each action step emerged from and determined the topic of subsequent meetings. Due to the various school schedules of the youth, these collective narratives were held on weekends, with lunch being provided. Fourth, after all youth had shared their experiences, the groups discussed resources needed and next steps. This led to the invitation of a guest researcher who showed them technologies that could augment their data collection. This phase was disrupted and then ended prematurely by the social distancing requirements and dramatic changes in the routines of the families during COVID-19. During the 1st year of social distancing, we contacted nine of the autistic co-researchers or consultants by phone or in-person to follow-up, asking: (1) How does the sensory environment impact you; and (2) What do you hope will come out of this project? Four youths provided written responses, two youths provided video-clip responses, and field notes were taken of phone call or web interviews with two youth and one adult (Total feedback: 180 min) (Total: 660 min).

#### Sub-Study on Sensory Experiences and Meaningful Participation

For the sub-study, we identified significant experiences across PV-1 and PV-2 transcripts. Significant experiences are those moments which stand out from the everyday flow of experience and can be identified by shifts to present tense, use of metaphor, and heightened emotionality (Mattingly and Lawlor, 2000). KL provided the key analysis for PV-1 and MAC provided the key analysis for PV-2. MP conducted analysis alongside KL and MAC for coherency and triangulation of results. KL, MAC, and MP then conducted an inductive thematic analysis of significant experiences related to sensory experiences and their articulated strategies within specific contexts. Finally, we examined the significant experiences related to sensory experiences, mental health and participation, using the participants’ descriptors (*stress, anxiety, meltdowns, drained*), heightened emotionality (*hate*), and/or language (*insane*). Pseudonyms were used for all participants, apart from the adult autist who is an advocate. Exemplary quotes were chosen by consensus and the final themes emerged during the iterative analysis between the three academic researchers, with a lead co-researcher providing critical feedback (NM). The results are presented to keep the first-person perspective intact to keep specific experiences situated within specific contexts.

## Results

As much as possible, we used the self-identifiers used by the children and youth when they shared experiences (e.g., person-first or identity-first). These self-identifiers did shift back and forth during and between collective narratives and interviews and, thus, we use both terms within the manuscript. In addition, we use the concept of bodily-sensing (e.g., see Park, 2008) to foreground the situated and embodied nature of the autists’ experiences. We’ve used the autistic youth and adult own bodily-sensing terms that represent their topical areas of concern that emerged from analyses to structure the results.

### “Motion Madness”: Bodily-Sensing Experiences and Participation

The autistic children, youth, and adult shared bodily-sensing experiences of hurt and pain that occurred in every space of their lives, whether in institutional spaces (schools, universities, hospitals), everyday civic ones (shopping malls, restaurants), or private homes.

#### “Hurt” and “Pain”

The autistic youth in the participatory projects described sensory experiences in terms of pain. These painful experiences are as Breanna (age 10, PV-1) indicates, *whenever* they go somewhere: “Whenever we go somewhere, if there’s a really bright light, it hurts my eyes and my brain really can’t focus.” Kathy adds how her son, Karl (age 11, PV-1), would wear sunglasses at the Children’s Hospital “because the lights were too bright and they were hurting [his eyes].” For Breanna, the hurt is not limited to bright lights, but also comes with the smells in a local mall’s bath and body shop: “The perfume and the smell, it really hurts, like, it goes up to my brain and it really hurts me in my brain.” Victor (age 17, PV-2) adds, “Dogs barking or howling, it’s like glass shattering in my head. As for vacuum, it’s like shaking my brain multiple times because of the sound.” These painful experiences are an inescapable part of everyday life. They are, as Breanna underscores, “always something in the back of my mind, in every place I visit.”

For Cassandra (age 22, PV-2), an older youth mentor, the technology-related sounds that pervade her classroom at university are painful:

*Everybody is on their laptops typing at the speed of light and that tikatik noise, which drills into my ears to the point where I could not focus on anything the teacher was saying ‘cause all I could hear was the noise of the other students around typing, and I would have [panic] attacks. I would walk outside of the class because that sound of people typing on their keyboards.*

The incessant sound of typing on keyboards is a *drill* so intense that she has *panic attacks* and must leave class. For Casey, an adult mentor (PV-2), it’s all the sounds in outdoor public spaces:

*I get overstimulated very easily. Like, just on the street with the cars, sirens, people, just the sound of everything. Especially awful noises, I am extremely sensitive and […] very high and low frequencies that other people can’t even hear are often really painful for my ears.*

As an adult mentor in the group, he offers a nuanced explanation of how the frequencies that others cannot hear causes the pain.

#### “Motion and Commotion”

For the children in the first photovoice study (PV-1), the biggest barrier was neither articulated in terms of discrete sensory systems nor multiple sensory systems. Rather, as the parents discovered in one conversation, it was the overall experience of *too much* of everything going on that was one of the biggest barriers to their children’s involvement in a life situation. For example, one of the parents described how her son Karl (age 11) would sit in the top near the handles of the grocery cart:

Kathy: Karl has to be in a cart because he can’t have anyone touch him. And he has to sit on the top because if he sits on the bottom, people might bang into him as we’re grocery shopping. And there’s just way too much motion and commotion going on. If everyone walked in a clockwise motion, I think maybe we’d be better, but-…

Saul**:** (a father of a preschooler with autism) -He fits?

Kathy**:** Oh, yeah. Trust me, we fit. Trust me, we even get our feet in there too. And what Karl does, if there’s too much motion and commotion going on? He shuts down and goes to sleep. Gone. Done.

Nina: It’s funny. Do you say, “motion and commotion,” or does he?

Kathy: I call it “motion and commotion.”

Nina: Okay. Because Victor calls it “motion madness” […] that’s when Victor would say, “that’s motion madness. I’m not going there, it’s motion madness.” He made it up.

Kathy continues saying that Karl will not go on the city bus either, because of the sound, smells, and “all the motion and commotion of people.”

More recently (PV-2), Victor found that online platforms used to learn during COVID-19 helped alleviate school’s motion madness, stating, “I like the gallery view so I can see everyone instead of the screen changing every time someone talks. It’s too much change.” Yet, eating out remains a challenge since “restaurants with multiple TV screens are stressful and distracting,” adding that “There is too much going on.” Casey (PV-2) is quite articulate about how the bodily-sensing experiences that Victor links to *madness* not only impacted his involvement in his work internship at a laboratory in the pathology department of a hospital, but also his mental health: “There were so many noises and the talk and other people. [….] it just like drove me insane.”

#### “All of a Sudden”

Victor’s motion *madness* and the layering of sounds and frequencies that drive Casey *insane* are amplified with the unexpected nature of sensory aspects of and in the built environment. This additional barrier to participation was reflected in a conversation between parents when their children were younger. Nina, Victor’s mother, remembers the challenge of public restrooms when Victor was about 8 years old:

*It isn’t that he doesn’t like toilets, but he hates the automatic flushers. He can’t stand that because some of them [motion detectors] are too sensitive. If he has to sit, he’ll jump up because he—and he’s not afraid he’s gonna get flushed down or anything—he just hates the element of surprise. ‘Cause some of them [motion detectors] are too sensitive and at the Theme Park, they have them. When we went to the Theme Park, he held his business for so long, ‘cause he’s like, “I’m not going in those [public restrooms].” And I said, “You just gotta go,” and sure enough, he did it and I heard him go, “Ah!”*

A researcher asks, “Oh, he screams?”

*Yeah, you heard him outside. He screamed. It scared him. And that was, like, three years ago. Now, he just avoids it. If he sees it, he avoids it. He’ll go somewhere else. He can’t stand it. He just doesn’t like these things.*

Two other families also concurred that they had the same experience, with Paul (age 11, PV-1) and Breanna (age 10, PV-1) underscoring the automatic nature and sound of hand dryers.

Cassandra, in a separate interview, links sensory sensitivities to the added impact when something happens *all of a sudden*:

*Especially with sensory sensitivities. I used to notice a lot of the time, if it happens all of a sudden-, if I’m not expecting this place to be really loud or I’m not expecting this smell, it will affect me to such a great degree. […] Obviously, the thing with sensory sensitivities is the more overwhelmed you are, the more anxious you are, depressed or frustrated.*

When the level of sound or smell exceeds Cassandra’s expectations or *overwhelms* her, the ultimate impact is on mental health (*anxiety, depression*, and *frustration)*. The experiences of the autistic youth and adult underscore the tight entanglement between bodily-sensing experiences of hurt and pain, mental health (*madness, insane, overwhelm, anxiety, depression, and frustration*) and any semblance of participation or involvement in a life situation.

### “Squishing” and “Squeezing”: The Layering of the Sensory-Social

Casey’s (PV-2) experience of being *driven insane*, situated within a work internship in a pathology department at a hospital, also underscores how painful bodily-sensing experiences are inextricably linked to social and built environments.

*There were so many noises and the talk and other people and then for certain chemical reactions, they had these alarm clocks, and then the buzzers went off and then the telephone, and then on top of all the machines-, on top of that, people really like to turn on the radio-, yeah the radio. It just like drove me insane.*

The layering—*and then, and then, and then, and then on top of all, on top of that*—drives Casey insane. For the youth and their parents, the layering of the sensory-social was described as the source of stress, pressure, and feeling trapped.

#### Stress

Nina (PV-1) described the challenges she faced when she had to take Victor, when he was younger (∼age 12), to the drop-in clinic for a recurring illness:

*Every time he has strep, which is, like, every month, we have to go to a drop-in clinic. Often a lot of public waiting rooms have these things where you’re face to face, like you’re sitting so close together. So he doesn’t like it because everyone is just staring at each other, so you’re trying to look away. He can’t stand that.*

When he is older, Victor (age 17, PV-2) more fully describes what he could not stand from his first-person perspective:

*Hospital waiting rooms are the worst […]. You are forced to be in the tiny room with many people, lots of smells, noises, babies crying, kids jumping everywhere, too much. I get really stressed.*

Ultimately, it is not just having to look directly at others, but the additional layering of smells, noises, and the motion madness of *kids jumping everywhere* that leaves him *really* stressed. The stress of the sensory-social layering also occurs when he uses public transportation:

*…very busy and crowded transport, you have many people squishing and squeezing you. It’s noisy and smelly and way too many people. It’s stressful. I cannot handle the busy of people. It gets so bad sometimes I cannot get off the bus or metro and I can miss my stop.*

The *squishing and squeezing* from, what he calls, *the busy of people* creates so much stress that Victor remains, literally, frozen.

*Many times, I have to get off at a different stop and walk because it’s too busy. I often leave 1–2 hours before and get up really early so I don’t need to be with so many people.*

He either ends up walking further or leaving one to two hours earlier. However, there is a price.

*But then I need to wait in the cold outside until I can go [in] to school. It’s too early, and they don’t let us in that early, and it is freezing outside. But it’s better than a stressful squished metro ride.*

Having to choose between a *stressful squished* metro ride and walking further or waiting in the cold, Victor bears with the latter. Not going to school is not an option. As Sophie (age 26, PV-2) explains, she often has “no choice” but to wait at the bus stop, despite the smell of someone smoking and “It’s not easy.” Casey’s experience of public transportation provides some additional details:

*If you’re in a metro and you don’t like to be touched? Well sorry, you’re never going to be on there at rush hour. It’s like sardines, right? So, it’s really hard. If you can at least get a chair, it’s going to help, because you have less people and their bags squishing you.*

Casey describes being squished during rush hour in terms of a can of sardines and the sense of touch as unavoidable. He tries to describe this experience for the non-autists in the group using sensory processing terms:

*Especially in the summertime there’s a lot of smells on the busy metro and the buses and so on. The sound and just the visual, it’s chaos. It really is like-, for you who don’t have autism or sensory processing [challenges] that don’t even feel that stress-, it’s like I get stressed out every time I go in public transit. I just kind of suck it up, but I hate it and I’m drained.*

In the end, Casey just has to *suck it up*, an effort that is tied to intense emotions (*hate*) and leaves him feeling empty (*drained*). This experience of public transportation is so overwhelmingly shared by the others that it leads to one of their hoped for actions (described in section “I Can’t be the Only One: From Individual Tricks to Universal Design”).

#### Pressure

Among the autistic youth and adult, the everyday pressure of time and crowds amplified the sense of pressure in their bodily-sensing experiences. For example, Paul (age 17, PV-2) recalls his experience of getting around crowded hallways at his high school as *hell time*: “For me, getting around the hallway was an absolute hell time because you have 5 min to maneuver up to halfway across the building, ending up being in crowded hallways.” Victor also describes how the mix of *too much going on*, the press of others in the small space of a locker room and time constraints adds to the experience of stress:

*Locker rooms are really busy, noisy. There is very little space-, very stressing, too busy, too much going on. It is a small place and we are pressured to rush and get dressed fast for gym. Lots of noises, weird smells, and people pressing against you.*

This double sense of “pressure” and “pressing” also occurs in situations in which time is not a factor:

*Bathrooms are really busy, like a stadium. It’s really stressful, there are too many people and too much noise from hand dryer, faucets, toilet flushing, line up people are too close.*

Like Paul and Victor’s description of the pressure, Casey’s experience of the layering of noise and crowds augments situations that are *already* inherently stressful:

*It’s already stressful having to go get a blood test, but with all the noise and crowd-, it’s too much, I cannot take it. It’s really chaotic. And then you have to go to this desk to talk about that and then standing-, standing in line, I always find it excruciating.*

This doubly stressful situation is experienced as *excruciating*. Yet many of these situations—public transportation to get to school, walking from class to class, locker rooms for gym class, and blood tests–are not really optional.

#### “Trapped” or Opt-Out

Casey, reflecting further, questions if the experiences of *excruciating* might be attributed to his own lack of patience before interrupting himself: “I think it also has to do with patience-, with also feeling trapped standing in line in the crowd-, here it is … the more difficult it is to find.” In the end, Casey reasons that it is the experience of being trapped in a situation that makes it hard for him to *find* patience. For Cassandra, it’s often better to leave:

*I will leave early sometimes-, have to take a shampoo bottle, unscrew the top and like smell the shampoo bottle just to get the smell away or I’ll have to leave the house because the smell [of cooking] will be overpowering.*

Cassandra and Casey first interpret their response to the sensory aspects of a hospital and a home in terms of their own characteristics or choices (*patience, leaving*). Yet, they both interrupt themselves. Casey reflects on how *standing in line* leaves him feeling *trapped*, while Cassandra interrupts herself to reflect on all the times she has first tried to stay in place by smelling something stronger than the smells that bother her. Still, the smell of cooking is, ultimately, so *overpowering* that she ends up leaving her home. As Cassandra astutely points out, the sensory and social cannot be separated:

*You can’t separate the social problems from sensory problems because if you’re already stressed out because of a social situation, you’re going to be more susceptible to sensory overload.*

The spiraling effect of the stress of a social situation increases Cassandra’s susceptibility to *sensory overload*, making it difficult for Cassandra to envision what she can do in the future:

*I don’t think I’d ever be able to have a job. You know, like in food places, because I’m so sensitive to smell, or you know stuff like that. Or-, you know, I’m sure that whatever job I ended up having, involved in dealing with other people my age, it would stress me out because of the whole social aspect of things too.*

Having to deal with the social aspect of things and her sensory sensitivities raises important implications for what kind of work Cassandra believes will be possible. For the autistic youth and adult, the entanglement between, and spiraling effect of, the social and the sensory often left them with no other options than to feel *trapped* or opt-out prior.

### “When You Don’t Respond in the Correct Way”: Categorical Misunderstanding of Individual Strategies

The autistic youth and adult shared stories in which their actions were grossly misunderstood, often explicitly linked to others placing them in denigrating categories and barely tenable positions. For example, Victor recalls an event in the hallway outside the cafeteria of his school at one of the meetings that receives an immediate response from a peer:

Victor: Recently, someone called me the R [retarded] word…

Keith: (age 17) Ohhhh [expressing empathy]

Victor: Yeah…-, right in the hallway during lunch, where I don’t like it because [showing photograph of the hallway] everyone is like scootched together like a bunch of prison mates.

Keith: Ouff

Victor: I’m like waiting here and everyone’s behind me. Every time I try to back away from them, to stay far, they come closer to me.

Victor then tries to *stick up* for himself.

*So, then I decided it’s time to just stick up for myself. So when they were starting to get too close to me, when I was trying to have some room, I just turned around and said politely, “First of all, I would greatly appreciate it if you would give me some space, thanks.” They did, but then they did it [come close] again. I was like, “Ahem,” just to tell them to “go away.” Well, after as they continue to bother me, it’s like, “Man, I got to think of some other ideas.”*

He then tries another approach by standing next to the line while sharing his discomfort with the students to the side and behind him.

*When I got into the line, it was impossible because the line was already fully lined up. So, I just stood next to it [the line]. But when I tried to get in, they wouldn’t allow me. They said I have to go in [the line] and I told them, “I don’t want to! […] It feels uncomfortable. It’s like being in a prison cell.” So then they told me, “It’s that or no lunch.” So, I did not eat lunch that day.*

Despite all his efforts—the polite request and thank you, the cue (*ahem*), standing next to the line and explaining the situation to his peers—Victor’s experience of being caught in a situation amplifies. Between enduring being called something unnamable (*the R-word*) and being in *prison* or not eating lunch, he feels he has no option but to take the latter.

Like Victor, Cassandra tries to find ways to remain in places she would like to be. Yet, often, these individual attempts are not enough. She gives an example of being in the classroom in which her attempt to remain in class is misunderstood:

*Sometimes I would wear noise-canceling headphones in class. The teachers look at me like, “Are you not listening to me?” or something like that. “No, on the contrary, I’m wearing the noise-canceling headphones so that I can listen to you!”*

Her actions are not just misread. They are grossly misunderstood and criticized. Such experiences are not limited to public spaces such as schools, but also at home with her parents.

*I would say, for me, my biggest sensory sensitivities are sound and smell. So the visual doesn’t bother me as much. Obviously, if there is a lot of like flashing lights and a lot going on, it will bother me. But definitely not to the point where sound and smell will, for example, when I’m at home and my mom’s doing something as simple as, you know, cooking a meal. Sometimes, it will cause a panic attack and my parents will not understand, “What’s the big deal we’re just cooking something in the oven.”*

The critique from persons in authority at university (*Are you not listening to me*?) along with intimate others at home (*What’s the big deal*…*.?*) underscores the impact that occurs when the very intentions underlying actions to stay in place are misrecognized. For Cassandra, this also includes being categorically misunderstood by her peers*:*

*The combination of loud music and the smell of alcohol and the smell of people smoking or whatever people are doing and the smell of food-, … the smell of this-, this sound-, people talking, you know, I have to put my foot down and I have to say, “Yeah, you know, I’m that weird girl who’s never gone to a party.” I will never go to a party, and that’s just something that I do because’ I don’t want to end up running out of the party and having a panic attack.*

Even when the youth attempt to stay in a place, like Victor and Cassandra, their actions were categorically misunderstood (*R-word, weird*).

Victor and Casey both speak about their sensory strategies in terms of *tricks*. Yet, Casey further delineates that such tricks can only be used in situations in which they are *socially accepted.*

*In public transportation, I have my tricks and listen to music or my mp3 player. But if you’re traveling with someone, then it’s not really socially accepted to be listening to music. When [I’m] communicating…. I can deal with it, but it would like drain me really fast and it’s everywhere. It’s everywhere I go. It has an effect. It doesn’t matter where you go, it’s everywhere.*

In order to be socially accepted, Casey must forgo using his *tricks* (listening to music) and communicate, an experience that *drains* him *really fast*.

For Casey, this double-bind experience is *everywhere* and can have even more dire implications:

*I was being accused of stealing something. And then-, when you don’t respond in the correct way or so according to them, then you’ve come across as even more of a threat. Then, I lose my ability to communicate in stressful situations like that and so-, so I cannot really tell them I am autistic. I just lose that ability.*

As an adult, *not responding in the correct way* can be perceived as a *threat* and lead to accusations that could put one in an actual prison. Worse, the stressful situation makes Casey lose the very ability to speak, by which he could alleviate misunderstanding and defend himself. Even more, he loses his identity as an autist, which is integral to his advocacy work.

The apparent choice between being categorically misunderstood when one doesn’t respond in the right way (*R word, weird, threat*) or foregoing lunch, having a panic attack, or being drained by the effort to be socially accepted is not really an option. In the end, the autistic youth and adult’s intentions to stay involved in a life situation using individual tricks (wearing noise-canceling headphones, standing next to the line, and listening to music in the presence of others) were categorically misunderstood.

### “I Can’t Be the Only One”: From Individual Tricks to Universal Design

During PV-2, the youth co-researchers and autistic adult also shared experiences about what was or could be useful in their everyday environments. They envisioned actions that could be taken toward a more hopeful future that, as they underlined many times, could also benefit others.

#### A “Quiet” Place

The youth and adult expressed the need to have a place to retreat. Cassandra voices that sometimes just a bit of preparation in a quiet room would be useful:

*But if I’m in a quiet room, getting ready, and I tell myself, “Listen, I’m going to be faced with sounds,” that “I’m going to be faced with smells” and I mentally prepare myself beforehand. Then, you know, it’ll be more effective.*

She continues that the quiet room or retreat could also help her recover from meltdowns caused by sounds and smells:

*I remember so many times having meltdowns. And if there was-, if there was that opportunity to have a room that I could sit there, even if it’s just a tiny room where I could close the door and be with myself and it would drown out the sound a little bit.*

Any space could be useful, even a *tiny* one. Any time could be useful, even for just *a little bit.* Such a space would not necessarily be devoid of any sounds or smells:

*I like it [the music room] because my music teacher has a pet bird, two guinea pigs and two rabbits, There are live animals. I love animals and they make me feel calm. The room itself is quiet, except noises from the animals (Victor).*

What Victor’s experience delineates is that it is the social and built environment rather than a natural one that is often the cause of stress and that connecting with animals brings a sense of *calm.* Ultimately, it is also the unpredictability of the sensory-social that concerned the autistic youth and adult. As David (age 17, PV-2) underlined, its more about knowing in advance the type of environment and how one can prepare in advance. He longed for an app that “if it existed, to be able to use when we plan an outing.”

#### Sensory Friendly “Zones”

To be able to prepare for a sensory-social environment using a quiet room or an app, however, would not fully rectify barriers to participation. The autistic youth and adult agreed that designated zones were needed across all everyday spaces. As David (age 17, PV2) best expressed, “more research [is needed] to continue to learn more and find ways to help and to advocate [….] talk to these commercial places, like restaurants, to let them know about sensory friendly zones.” The term, *zones*, emerged during the discussions during PV-2 as a critical modifier to the more common phrase of sensory friendly. After debating the relative merits of space or zone, they agreed that not only were *zones* within institutional and everyday public spaces a more achievable aim but one that would not separate them from others. They also reasoned that a *zone*, integrated into already existing spaces with proper signage, could also include others seeking a similar sense of quiet.

For Cassandra, a publicly accessible sensory friendly *zones* would align with her desire to educate others about autism:

*I always dreamed of, you know, putting myself in a place where I know there’s a lot of students and just handing out like things educating people on what autism is, you know? People that work in places like restaurants or even, you know, police officers, the security guards in malls, for example.*

She imagines that if others understand the relationship between the sensory experiences in social, built environments and mental health, then others would also benefit:

*I can’t be the only one who has meltdowns in malls or meltdowns in a train station. Sometimes all it needs is, you know, one security or two tops that has some knowledge and some awareness of-, they can make a training, they can train people, it’ll take them maybe two hours to explain to them. This is, you know, this is autism, this is mental illness, this is what you do, this is what you don’t do.*

Cassandra’s words, “I can’t be the only one,” stress the inclusive nature of her reflection. If the staff of different public services such as transportation, restaurants, police forces, and policy makers were trained to understand *what you do* and *what you don’t do*, many others would benefit.

#### “People Just Know”

The autistic youth, their parents, and autistic adult all indicated across both studies that training or educating others was not enough. Although there were many stories of when someone who was educated or understood made all the difference for their experiences—whether a police person, a hostess at a restaurant or staff at a water park—what was needed was a universal sign. They all desired that such a universal sign could, for example, be placed in all public transportation vehicles to designate a sensory friendly *zone*, whether actual seats or just a place to stand. For Cassandra, such signage could help with the process of training: “I feel that the use of a new disability symbol with help with that process immensely since it will show that there are more disabilities than just physical ones.” For Casey, such signage would also help others understand his actions:

*I wouldn’t mind that something would be there that you can-, like, it’s in your card, or where people just know that you have autism. And I also find, at least, like dealing with cops, and security guards, they noticed something about me that’s unlike-, more on edge or a bit nervous or maybe. It will be nice if I add something on my card and I can point to in order that, “Oh, this person is autistic, is not a threat.”*

Although Casey is talking about having a card that he could point to so that others *just know*, the youth wanted a symbol and a sign to do this work. A public, universally recognized symbol would not only alleviate being immediately labeled as a *threat* on an individual level, but could also become inclusive of and provide a sensory friendly *zone* for others.

In the end, the youth with ASD and their parents agreed that a universal sign should neither categorize them as disabled nor suggest they be protected (*safe*), ultimately opting to use an existing phrase in the public sphere of sensory friendly and modifying that by *zone.* They envisioned a symbol that would not segregate persons but one that could be used within and include others in already existing spaces. Victor best articulated the inclusive nature of their shared vision, stating, “I hope all the mayors learn from this so they can make their cities more sensory friendly.” Victor imagines what could happen if all mayors understood the impact of social built environments on bodily-sensing experiences and how the creation of sensory friendly *zones* could create more inclusive cities.

## Discussion

In the following section, we discuss how close attention to the experiential knowledge of autistic youth, their parents, and autistic advocates of their own bodily-sensing could inform concepts about participation used in rehabilitation and approaches to mental health while opening up new areas for relational approaches to research and social transformation.

### Remapping the International Classification of Functioning, Disability, and Health: First-Person Perspectives of Bodily-Sensing and Participation

Taking the first-person perspectives of autistic children and their families (PV-1) as well as autistic youth and adult (PV-2) into account clearly illuminates how the sensory-social is indelibly and inextricably linked in experience and impacts on involvement in life situations. Yet in third-person perspective research, social and sensory constructs are often examined as separate factors. Further, third-person perspective research still locates deficits within the individual. For example, Askari et al.’s (2015) scoping review notes how participation of children with ASD is impacted by “the core deficits of an ASD [diagnosis] (i.e., communication impairments, social deficits, and abnormal restrictive, repetitive, and stereotyped behaviors) as well as other characteristics associated with the disorder (e.g., maladaptive behavior)” (p. 112) with only a relatively scant amount of research suggesting that social support and negative attitudes were central factors. In contrast, a focus on sensory processing leads to different implications. For example, Dunn (2007) suggested that her model of sensory processing could be used to adapt everyday life situations—particularly activity contexts—to support the needs and participation of children with different patterns of sensory processing. Ismael et al.’s (2018) systematic review, which specifically focused on research that used Dunn’s sensory processing framework (Dunn, 2001), found that the sensory processing of children with ASD impacted their participation and concluded by suggesting that the sensory environments of activities are critical factors for participation.

In our participatory project, we used the Short Sensory Profile (SSP) (McIntosh et al., 1999) and the Adolescent and Adult Sensory Profile (AASP) (Brown et al., 2001) to focus on sensory experiences rather than as a measurement. However, the scores did show that the children with ASD in PV-1 presented higher scores in the visual/auditory filtering categories with patterns of under-responding/sensory seeking. The sensory sensitivities of the autistic youth in PV-2 who had participated in PV-1 remained the same in the AASP. Yet their scores in PV-2 also showed changes in their related sensory patterns (from *sensory seeking* to *sensory avoiding*). On one hand, this suggests that sensory sensitivities alone did not impact participation. For example, they shared experiences that clearly demonstrate that they had all developed individual *tricks* to stay in or travel to spaces in which they wanted to do valued activities that were meaningful. Instead of individual deficits as barriers to participation—related either to the diagnostic criteria of autism or sensory processing—the most incalcitrant barriers to meaningful participation were the social responses (or lack thereof). Based on specific, situated and embodied experiences, the autistic youth and adult’s individual *tricks* led to being categorically misunderstood (*R-word, weird*) or *drained* by their attempts to *respond in the correct way.* On the other hand, this also suggests that being categorically misunderstood and drained by efforts to do what is *socially accepted* could also shift sensory processing patterns from seeking to that of avoiding.

#### Making the Layering of the Sensory-Social Visible

The autistic youth and adult experienced the social and built environment in homes and institutions (*smells, bright lights*, enclosed spaces, and placement of chairs) and it’s layering in everyday objects (*alarms, vacuums, buzzers, sirens, telephones, televisions*, and *machines*) as *excruciating, overwhelming*, and the cause of *hurt* and *pain*. Their concrete suggestions for change, such having a quiet space, mirrored those of the autistic authors in Davidson’s (2010) review who suggested “toning down ‘toxic’ stimuli – such as fluorescent lights” (p. 305). Yet the domains of the International Classification of Functioning and Disability (ICF) as currently mapped could neither lead to such existential perspectives nor capture such concrete suggestions for social-spatial inclusion.

Rather, the ICF domains constrain research to a limited range of, and very different, causal factors impacting on participation. For example, no studies in the scoping review of sensory processing and participation of youth with ASD investigated a comprehensive range of determinants (Askari et al., 2015). What the studies did cover were different domains, such as: environmental factors (family support, social attitudes), body functions (sensitivity and behavioral challenges), and activity limitations (communication and interpersonal relationship problems). However, the environmental domain only focused attention on material, natural and social environments without any explicit indication of its sensory aspects. In addition, no interrelationships between domains were explored.

In the sub-study of the participatory project, the autistic youth and adult shared experiences in which bodily-sensing was inseparable from both social attitudes and the social built environment. However, it is their bodily-sensing experiences from their first-person perspectives that so clearly illustrates how the layering of the sensory-social limits their participation to feeling *trapped* with the only options being to *suck it up or leave*. In a similar vein, Davidson’s (2010) review of 45 autobiographical texts showed how autistic authors’ “extraordinarily heightened senses” were barriers to social-spatial inclusion (p. 305). Davidson used the term “sensory geographies” to illuminate how persons must navigate physical environments that are also social spaces that contain, what she calls, “sensory furniture.” She notes that even though sensory furniture could easily be moved, it is not. This inflexibility decreases the possibility of access as well as the sustainability of participation across time. Like Davidson’s sensory furniture, the autistic youth and adult in the participatory project underscored how it was the social built environment which led to *hurt* and *pain* and eventually, their leaving (even home) or opting out entirely. Davidson’s background as a social geographer, however, points out the potential and universal implications of the autistic youth’s participatory project in their directives for what new furniture to add—in the form of signage (sensory friendly *zones*)—and what to dismantle entirely.

#### Threading (Agentic) Meaning Throughout

The focus of the participatory project on bodily-sensing experiences supports an ongoing discussion about how the ICF could be restructured to better map subjective experiences of participation. As Mitra and Shakespeare (2019) note, keeping health conditions at the top of the diagram and positioning personal factors below and separate from environmental factors raises critical questions, such as: “What about the agency of the individual?” and “What if the activities under consideration are not those that are valued?” (p. 338–339). Thus, Mitra and Shakespeare (2019) suggest moving personal factors to the top of the diagram and moving health conditions below. The bodily-sensing experiences of being *squished, squeezed*, and *trapped* in everyday spaces by the children with autism and youth co-researchers underscore how their choices are limited and constrained by the layering of the sensory-social. One simple example is how, from a third-person perspective, Casey, Sophie, and Victor use public transportation to participate in things that matter to them. Yet, on closer inspection, the layering of the sensory-social limits their sense of agency by determining when they can travel if they want to avoid such experiences (Victor) or resign themselves to being drained entirely (Casey). The third option, of course, is to simply opt out. Although only descriptive, it is notable that the sensory processing patterns of the three youth who participated in PV-1 and PV-2 do show a shift from sensory seeking to sensory avoiding.

Jahiel’s (2015) reconceptualization of the ICF marks how focus groups and interviews led to the development of subjective measures of meaning and values while ethnographic accounts of the experiences of disabled persons that highlighted the significant impact of environmental factors on participation did not subsequently lead to new measures. Jahiel attributes this gap to the separation of the personal from environmental domains in the model, arguing for the reformulation of the personal-environmental as more interactive and dynamic than depicted. The autistic youths and adult’s bodily-sensing experiences of the impact of the *sensory-social layering* on their participation certainly buttress this argument. In Jahiel’s reformulation, one new domain of “intent” is created with all the domains moving to the same level to visually emphasize the interactional or even transactional quality between them. Placing all the domains on the same level, while also making intentionality a distinct domain, places the environment on equal footing as agency. This could also highlight the important work that parents already do to facilitate their children’s agency and control over their environments through modifications and-or advocacy (Pfeiffer et al., 2017).

Two concepts, “scene setters” (Badley, 2008) and “scene setting” (Jahiel, 2015) could accentuate how the value-laden aspects of the environment impact on existential experiences tied to participation. For Badley (2008), scene-setters refers to societal contextual elements that influence participation, which could account for and make visible the constraints of sensory-social layering on persons’ full involvement in a life situation. By extension, the “scene-setting aspect of the environment determines what certain aspects of functioning mean, what is relevant to us in a particular context, how we do things, and what […] options we have at our disposal” (p. 2,337). For Casey, the layering of the sensory-social in his work internship strips him of any sense of agency (*drives him insane)* and determines its meaning (*hate)*, which provides nuance. Persons may appear to be fully involved in a life situation from a third-person perspective but may be doing so in spaces where the societal-contextual elements negatively impact on their experience. This has important ramifications for, as will be pointed out below, mental health.

The longitudinal nature of the participatory project also provided an embodied, situated and temporal view of participation from first-person perspectives. As it has been pointed out, the ICF is static and understanding participation and developing adequate measures will require much more than cross-sectional research (Askari et al., 2015) that rely on snapshots in time (Jahiel, 2015). Victor’s experience in the lunch line and Cassandra’s experience at school, for example, provide rich descriptions of how what may look like involvement in a life situation can change from minute to minute. From a third-person perspective it may appear to a casual observer that both Victor and Cassandra are participating in school and university activities respectively. Yet, despite their efforts to stay in line (explaining to peers) or class (wearing noise cancelling headphones) both opt-out in the end (not eating lunch, leaving class).

A recent definition of *occupational* participation could prove useful to help delineate the range of entry points toward evaluating participation temporally. In occupational science, the concept of occupation is defined as the ordinary things that persons do that really matter to them, and in which “the meaning or lack of meaning to occupations” (Yerxa, 1990, p. 9) can only be revealed by an individual’s experience of them. Thus, to some degree *occupational* participation provides another way of conceptualizing meaningful participation, albeit from a first-person perspective. Further, occupational participation is defined as “accessing, initiating, and sustaining valued occupations within meaningful relationships and contexts” (Egan and Restall, 2022). Although Victor and Cassandra are able to access and initiate valued activities, the categorical misunderstanding of their actions on top of the layering of the sensory-social is just too much. They are unable to sustain their participation across time.

### Socio-Spatial Inclusion: The Mental Health Costs of Being Socially Acceptable

The autistic youth and adult in the participatory project made clear links between their lack of control over the layering of the sensory-social and their mental health. They reported on how the painfulness of the sounds, smells, sights, and motion-commotion—and its unexpected nature—in the social and built environment was, as Victor explains, *madness.* However, these metaphorical descriptions have real implications. In her ethnographic study, Bagatell (2007) described how the layering of the sensory-social leaves Ben, a 21-year-old college student with Asperger’s, more and more depressed until one day he finds himself sitting on a windowsill with his feet dangling over the edge:

*They’re feelings [panic] that just get too powerful. It’s not so much feelings like emotional feelings but it’s like physical, physiological. […] It’s like outward pressure. […] It makes me crazy sometimes. I just don’t know what to do (p. 423).*

The panic attacks that Ben experiences in his struggle to orchestrate the voices from, what he calls, the “Aspie” world with the everyday one are less about being an emotional state than a physical and physiological one. His bodily sense of *outward pressure* aligns with the autistic youth’s experiences in the participatory project of the *pressing* of others and *pressure* of time. For Ben, the outward pressure *makes* him *crazy*, much like the layering of sounds and their frequencies *drives* Casey *insane*. For Ben, this sensory-social experience reaches an existential breaking point in which he can only envision a life alone, which leaves him questioning, “What kind of a life is that?”(p. 424).

Despite the extensive neuroscience research on the relationship between atypical sensory processing and psychopathology (e.g., see the review by Bailliard and Whigham, 2017), there is a surprising lack of attention to the relationship between actual pain, unexpected or unanticipated aversive input, lack of agency in particular contexts, and mental health. In their research of sensory processing patterns in the general population, Dean et al. (2018) found that sensory seeking was negatively correlated with depression, suggesting that sensory seeking could be a positive predictor of resilience and adaptability. More specifically, children who actively engage in their environment demonstrate fewer signs of depression and are more likely to be resilient. They also suggest that children who, on the contrary, are more avoiding would gain from interventions that would assist them in developing self-regulation strategies viewed as “appropriate to their peers, parents, and teachers” (p. 6) to support participation. Yet, the autistic youth and adult in the participatory study underscored that it is the double sense of pressure—navigating the sensory geographies and the social expectations of what is acceptable—that left them *drained* and categorically misunderstood in their schools, communities and workplaces. Their continuous efforts to *respond in the correct way* ultimately empty them in ways that are, as Ben’s experience also hints at, potentially world ending.

#### How Does One Proceed?

Speaking of her own experience, the autist and anthropologist Prince (2010) says:

*Since I can remember—and that is from my own beginning—I have been pierced and pained by the intensity of life. There were many times as a child I believed I would crumble in on myself, my emotional skeleton finally eaten away by the screaming and clutching of a modern society that dissolved me—normal life, other people call it (p. 56).*

The intensity of the “normal life” *crumbles* and *dissolves* her much like the layering of sensory-social (*on top of, on top of)* renders Casey speechless, taking away his ability to disclose who he is as an autist—and, thus, his own identity as an advocate and activist. For Prince, the *piercing* and *pain* are too much, and she quits school at an early age, becoming homeless for several years. She explains, however, that opting out was not about not caring but precisely the opposite. As a “naturally connected person” (p. 56), she cares (almost) too much, a characteristic that she observes in her son’s interactions moving bugs that are so tiny they are barely perceptible to a place where they would not drown in their back yard.

His natural connection to everything around him not only left him vulnerable to others’ judgements but also the to the lights that start to hurt his eyes and “the normal noise of conversation [that] hurt his ears[…]” (p. 65). Prince’s descriptions of her son’s experiences are also reflected in the *hurt* and *pain* that the children with ASD, autistic youth and adult in the participatory project attributed to bright lights, sounds of buzzing, alarms, music, talk, engines, hand dryers—especially when they occurred *all of a sudden.* Prince’s son, “… would cover [his ears] with his hands and rock, trying to get under the table,” what she calls, being “contextually autistic” (p. 66). When she took him out of school and provides home schooling, he flourishes.

In addition to its impact on participation, the pressure to *respond in the correct way* and be the person one “should” be to “fit in” (Bagatell, 2007, p. 417) or to “pass as normal” (Prince, 2013, p. 329) has larger repercussions for mental health. For Cassandra, the navigation of her sensory sensitivities coupled with the *social aspect of things* raises critical implications for what she believes might be possible for her. When individual strategies fail, are dismissed and-or categorically misunderstood, what are the real options?

Although the anthropologist Prince (2013) points attention to how societal contextual factors come to define the kind of person one is, she is also making a larger claim as an autist: Any attempts for autists to *pass as normal* create barriers to three universal desires held by all people:

*The ways we pass as normal keep us from having any of our three deepest wishes granted like heaven; we can’t be loved for who we are, because we hide ourselves, knowing we are freaks; we can’t give, because we are often too afraid; and because no one knows who we are or what we can give, we are afraid to die, knowing we can’t truly be remembered (p. 329).*

If one is only something unnamable (*R-word*), *that weird girl*, a *threat*, or a *freak* than one has not existed at all. As Prince profoundly points out, the very actions that allow one to *pass as normal* are also the very actions that do not allow one to be fully seen. The very actions that allow one to pass as normal are also the existential barriers to participation at the deepest levels, in which social expectations and standards about what is normal render one virtually invisible where *no one knows who [one is] and what [one] can give*.

This shifts the question from “how does *one* proceed” to “how do we, as a society, proceed?”

#### A Relational Approach to Sensory Curb-Cuts

In geographer and critical autism studies scholar Davidson’s (2010) relational approach, she draws on an aggregate of autistic authors’ insights about social-spatial exclusion. Such first-person perspectives are necessary, Davidson suggests, to re-imagine sensory geographies since sensory experiences are often hidden from and inaccessible to others. The concept of “relational” is used in a commonsense way as based on mutual understanding (p. 306). A relational approach foregrounds being sensitive to others’ ways of being and the reciprocal responsibilities toward one another when occupying spaces together. Thus, re-imagining sensory geographies in such a relational approach entails listening deeply to what autists share and taking responsibility, as a society, to redesign the spaces we inhabit together.

The autistic youth and adult of this participatory study give a clear vision of what can be done. For them, the space that is needed is simply a *zone*. A place not just where they can participate, but one that is equally friendly to others. As Cassandra reflects, “I can’t be the only one who has meltdowns.” In universal design principles, the curb-cut in sidewalks is the most common exemplar. Once cuts were made in sidewalks to support the accessibility of persons in wheelchairs, they also immediately became useful for a variety of others—whether for baby strollers, toddlers, older adults, those with visual impairments or simply those who prefer to shuffle. Cassandra also alludes to the potentiality of not just considering physical barriers in her reminder that there are “more disabilities than just physical ones.” Listening deeply to persons with ASD and autistic persons could expand sensory accessibility for all.

Dunn et al. (2012) proposed a “contextually relevant” reflective guidance for occupational therapy interventions linking sensory processing principles to a family’s routines and settings so that parents can learn about how their children’s sensory processing patterns might affect their participation. The parents, then, become responsible for helping creating spaces for meaningful participation for their children. With this approach to modifying activity contexts and informing parents about sensory processing principles, the participation of children with autism aged between three and 10 years old increased. Pfeiffer’s (2017) research showed how parents support their children’s sense of agency and control over the environment in ways which increase their participation. The focus of these studies align with the relationship-centered focus in occupational participation (Restall and Egan, 2021). However, the weight of responsibility remains on children, their families and the health professional who work with them.

Cassandra’s vision, however, expands the potentiality of these efforts by shifting the responsibility to other social sectors. She suggests that it would only need “one security [personnel] or two tops that has some knowledge and some awareness of [autism], [so] they can make a training, they can train people.” Her vision could materialize, an approach in which we, as a society, “are willing to take relational responsibilities seriously and really hear” what autists have to say (Davidson, 2010, p. 311). When *the busy of people* becomes *too much*, having a space to go while not necessarily being alone is something that the autistic youth desired. For Cassandra, avoiding meltdowns is as simple as a quiet place, even a *tiny* one, where she can prepare herself. For Victor, it is a space to be *calm*, and even better if there are the sounds and presence of animals. Yet, what the autistic youth, their parents and the autistic adult mentor collaboratively agreed upon in their meetings was not a demarcated or separate space but integrated *zones* that are sensory friendly.

On several occasions, Nina brought attention to the book *Designing for Autism Spectrum Disorders* which aims to explain “how architecture and interior spaces can positively influence individuals who are neurodivergent by modifying factors such as color, lighting, space organization, textures, acoustics, and ventilation” (Gaines et al., 2016, p. 3). Creating social-spatial inclusive spaces on an even larger scale is best exemplified, in turn, by Victor’s response to the question, “What do you hope will come out of this project?” His answer gets right to the heart of a relational approach: “I hope all the mayors learn from this [project] so they can make their cities more sensory friendly.” Victor imagines what could happen if the mayors (plural) could learn from the experiential knowledge they shared. It’s a vision where others know who they are and what they can give. It is not just inclusive cities that they envision, but “mutually ‘inclusive’ societies” (Davidson, 2010, p. 311) that is Cassandra’s and Victor’s wish.

## Study Limitations

The sensory experiences are limited to the urban area in which the participatory project was situated. Future studies would benefit by listening to the experiences of autistic persons from multiple areas. In addition, the regulations associated with the COVID-19 pandemic interrupted group gatherings in March 2020, constraining our ability to take action steps as well as conduct thorough member-checking on the themes reported here.

## Data Availability Statement

The original contributions presented in the study are included in the article and any further inquiries can be directed to the corresponding author.

## Ethics Statement

The studies involving human participants were reviewed and approved by Research Ethics Office (IRB) of the Faculty of Medicine and Health Sciences. Written informed consent to participate in this study was provided by the participants’ legal guardian/next of kin. Written informed consent was obtained from the individual(s) for the publication of any potentially identifiable images or data included in this article.

## Author Contributions

MP designed PV-1. MP, KL, and the families collected and analyzed the PV-1 data. MP, NM, and AS designed PV-2, and collected and analyzed the data along with the youth and M-AC. M-AC, KL, and MP are co-first authors of the sub-study, contributing to the analyses of results and representation of the data. KL and MP revised and wrote the final manuscript. All authors read and approved the final submitted version.

## Conflict of Interest

AS was employed by company addtothenoise. The remaining authors declare that the research was conducted in the absence of any commercial or financial relationships that could be construed as a potential conflict of interest.

## Publisher’s Note

All claims expressed in this article are solely those of the authors and do not necessarily represent those of their affiliated organizations, or those of the publisher, the editors and the reviewers. Any product that may be evaluated in this article, or claim that may be made by its manufacturer, is not guaranteed or endorsed by the publisher.
